# Shear strength deterioration effect of rock mass joint surface under cyclic shear load

**DOI:** 10.1038/s41598-022-19385-0

**Published:** 2022-09-03

**Authors:** Heng Zhang, Shan Dong, Zhichun Lu, Yulin Peng, Weihua Hou

**Affiliations:** 1 Badong National Observation and Research Station of Geohazards, China University of Geosciences, Wuhan, 430074 China; 2 Three Gorges Research Center for Geohazards, China University of Geosciences, Wuhan, 430074 China; 3 CISPDR Corporation, Wuhan, 430074 China

**Keywords:** Civil engineering, Natural hazards

## Abstract

Understanding the shear strength degradation mechanism of a rock mass joint surface under cyclic shear load and determining a corresponding analytical model is an important foundation for accurately evaluating the safety of rock mass engineering under seismic loads. It is worth noting that, to date, there has been a dearth of studies on the strength characteristics of joint surfaces that consider the number of loading cycles, normal load, and initial undulant angle of the structural plane. In this study, focused on the behaviour of sandstone, the particle flow code (PFC) modelling framework was used to simulate a joint surface cyclic shear test considering first- and second-order undulations. Based on the experimental results, the comprehensive effects of the number of cyclic shear cycles, the normal stress, first- and second-order undulation and the dilatancy angle on shear stress during cyclic shear were analysed. Formulas for the joint surface shear basic friction angle and dilatancy angle under cyclic shear were proposed, and a method for calculating the joint surface peak shear strength under cyclic shear considering the deterioration of the dilatancy angle and basic friction angle was established. The peak shear strength of a sample after five cycles of shearing was calculated using the proposed formula and compared with the results of numerical simulations, the Barton method, and the Homand method. The results showed that the calculated values have good consistency with the results of the numerical simulations, demonstrating the effectiveness and accuracy of the proposed formula. However, under a low normal stress, there could be errors in estimating the cyclic shear strength of the joint surface.

## Introduction

Jointed rock masses are often observed in rock-mass engineering. The primary difference between a rock mass and intact rock is that the rock mass has many discontinuous surfaces (or structural surfaces), including faults, joints, and weak interlayers—and the existence of these discontinuities largely controls the properties of the rock mass^1–3^. Furthermore, the stability of rock slopes and other rock engineering projects is directly related to the shear strength of the joint surface, such as the bedding rock slope^4,5^. Under the action of a strong seismic load, a cyclic shear phenomenon across the peak shear strength appears on the interlayer structural plane, resulting in the degradation of the initial morphological characteristics of the interlayer structural plane and a decrease in the shear strength, directly affecting the stability of the slope. Generally, deep underground engineering rock mass has joints, fissures, weak layers and other structural planes, which often weaken its strength and stability; which are highly important for engineering safety^6,7^. On the one hand, under the action of tectonic stress, the rock will undergo relative dislocation along the joint surface, that is, shear action; on the other hand, under the seismic load or mining condition of the coal seam working face, the rock will undergo cyclic shear along the joint surface, further weakening the mechanical strength of the rock mass. Therefore, the study of the deterioration mechanism of the shear strength of rock mass joint surfaces under cyclic loading is a crucial issue to be solved to precisely evaluate the dynamic stability of a slope or deep underground engineering rock mass under seismic loads.

Most former indoor laboratory tests on the mechanical characteristics of rock mass joint surfaces have focused on defining the peak shear strength and stress–displacement relationship under uniaxial shear load^8–13^. However, there have been few studies on the cyclic shear of rock joint surfaces, with previous studies on the shear characteristics of joint surfaces being based primarily on indoor joint surface cyclic shear tests. Huang et al.^14^ tested sawtooth-molded joint surfaces under cyclic shear loading and proved the wear degradation law of joint undulations proposed by Plesha^15^. Based on the shear test results of 50 concrete samples of rock joints, Jing et al.^16^ put forward a conceptual model for the action of rock joints during cyclic shear and under a constant normal load. Divoux et al.^17^ summarized the results of multiple cyclic shear tests and proposed a cyclic shear mechanical constitutive model for a joint surface. Homand et al.^18^ conducted cyclic direct shear tests on an undulated manual structural plane and proposed a formula to forecast the degradation of anisotropic structural planes and cyclic shearing modes. Lee et al.^19^ conducted a series of cyclic shear tests on sawing and split tensile joint surfaces and put forward an elastic–plastic constitutive model that considered the degradation of second-order undulations. Jafari et al.^20^ carried out a research on the shear strength of a cubic sawtooth rock joint surface under cyclic shear loading and put forward a shear strength formula for a joint surface sample under the condition of a large cyclic shear displacement. Fathi et al.^21^ conducted a set of cyclic shear tests on artificially replicated crude granite joint surfaces and found that with an increase in the number of shear cycles, the roughness of the joint surface and shear parameters of the joint surface continued to decrease. Through a joint surface cyclic shear test, Mroz et al.^22^ analyzed the degradation of undulations during the structural plane shearing process and put forward a constitutive model that could effectively reflect the softening of the shear stress curve after the first cyclic shear. Although there have been some relevant studies, there have been few reports focused on the comprehensive physical and mechanical characteristics of rock mass joint surfaces under cyclic loading with repeated changes in the shear load direction.

Moreover, it is highly important to obtain the in-situ mechanical properties of rocks; however, in-situ mechanical tests are often hard to conduct^23^. Owing to the difficulty in sampling natural joint surfaces, it can be difficult to guarantee the consistency of each sample during testing. However, it has been proved that it is feasible to prepare rock structural plane samples with similar materials—in particular, the artificial serrated regular joint surface can quantitatively reflect the influence of the undulant angle on the mechanical properties of a joint surface^24–28^. Nevertheless, the physical model test can be expensive and time-consuming, and the deformation and failure processes in the shear process of the joint surface can be tough to view^29–31^. Consequently, numerical simulations offer a promising method to complement or even substitute physical tests. The particle flow code (PFC) model proposed by Cundall could simulate the adhesion and friction between rock mineral particles on a mesoscale, thereby avoiding the use of empirical parameters to set the macroscopic constitutive model. Today, it is extensively applied to the analysis of the mechanical properties of rocks as well as the shear test of rock joint surfaces^32–36^.

Several scholars have carried out particle flow numerical simulation tests on joint surface cyclic shear. Based on physical experiments, Xu et al.^37^ used a two-dimensional particle flow (PFC2D) model to conduct cyclic shear numerical simulations, studied the macroscopic and microscopic fatigue damage mechanisms of multi-scale micro-convex rock-joint surfaces under constant normal stress, and simulated the meso-fatigue damage evolution process of a serrated rock structural plane. Kou et al.^38^ conducted a particle discrete element numerical simulation to deeply understand the mechanical response of multi-scale triangular micro-convex (first- and second-order undulation) rock joint surfaces under pre-peak cyclic shear tests. Liu et al.^39^ used a PFC2D program to realize a pre-peak numerical test of cyclic direct shear of rock joint surfaces with second-order undulation and analysed the change law of joint cumulative damage characteristics and shear strength in the pre-peak cyclic direct shear test of joints from both a macroscopic and microscopic perspective.

In this study, numerical simulation tests of joint surface cyclic shear considering first-order and second-order undulations using a PFC2D model were carried out, and the shear stress–displacement curve was analyzed. Starting with the deterioration of the dilatancy angle and the basic friction angle, a formula for calculating the peak shear strength of a rock mass joint surface under cyclic shear load was proposed. It was then compared with the research methods of Barton and Choubey^40^ and Homand et al.^18^. It is hoped that this study will provide a fundamental theoretical basis for obtaining the shear strength of joints more precisely, benefiting the development of stability analyses of rock slopes or deep underground engineering under seismic loads.

## Methods: cyclic shear test of rock mass joint surface

**Figure 1:**
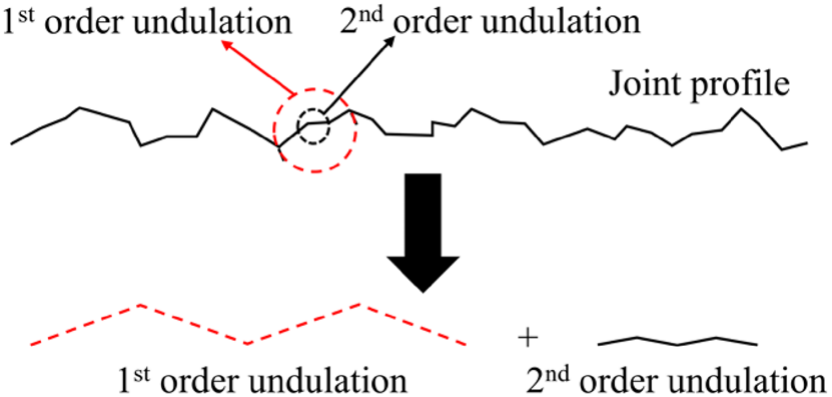
Conceptual model of first- and second-order undulation (diagram from Lee et al.^19^).

The real joint surface is composed of randomly distributed undulations. According to the different geometric characteristics of undulations, rock joints are composed of rough surfaces, and there are many micro-convex bodies from the macro-scale to the micro-scale. Lee et al.^19^ proposed that the morphology of a joint surface could be approximated using the superposition of first- and second-order undulations, as shown in Fig. 1. The first-order undulation is a large undulation (sawtooth and step) on the joint surface, which represents the undulating trend of the joint surface, and it is often used to represent the common peak valley undulating contour on the surface of the joint surface. The second-order undulation is the undulation with small size on the joint surface and represents the roughness of the joint surface. It mainly represents the secondary micro-geometric characteristics of the undulation on the joint surface. The size of the first-order undulation is approximately one order of magnitude larger than that of the second-order undulation.

Several important results have been obtained pertaining to the study of the cyclic load shear strength of joint surfaces considering only first-order undulations^41–44^. However, Belem et al.^45^ highlighted that the classic rock mass shear strength model did not consider the influence of second-order undulations, and underestimated the shear strength when the normal stress was low. Through cyclic shear tests of rock mass structural planes, Liu^46^ inferred that second-order undulations had a considerable impact on the shear characteristics of rock mass structural planes, particularly when the normal stress is small. Consequently, ignoring the second-order undulation significantly underestimates the peak shear strength of the joint surface.

### Parameter calibration

The meso-level parameters involved in PFC2D possess internal randomness and a complicated relationship with macro-mechanical properties. By simulating basic mechanical tests, it can be ensured that the macroscopic mechanical properties are consistent with the actual experimental results, and that the calibrated parameters can be considered reasonably^47–51^.

**Figure 2:**
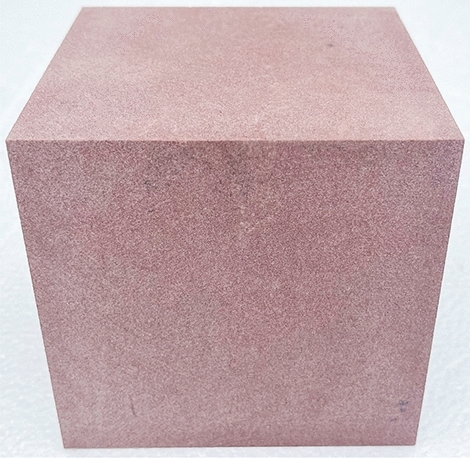
Sample without a joint.

**Figure 3:**
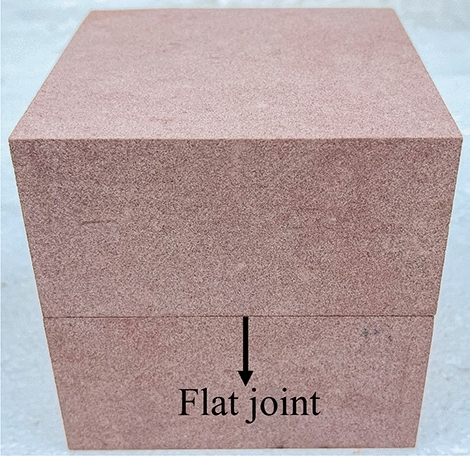
Sample with a flat joint.

**Figure 4:**
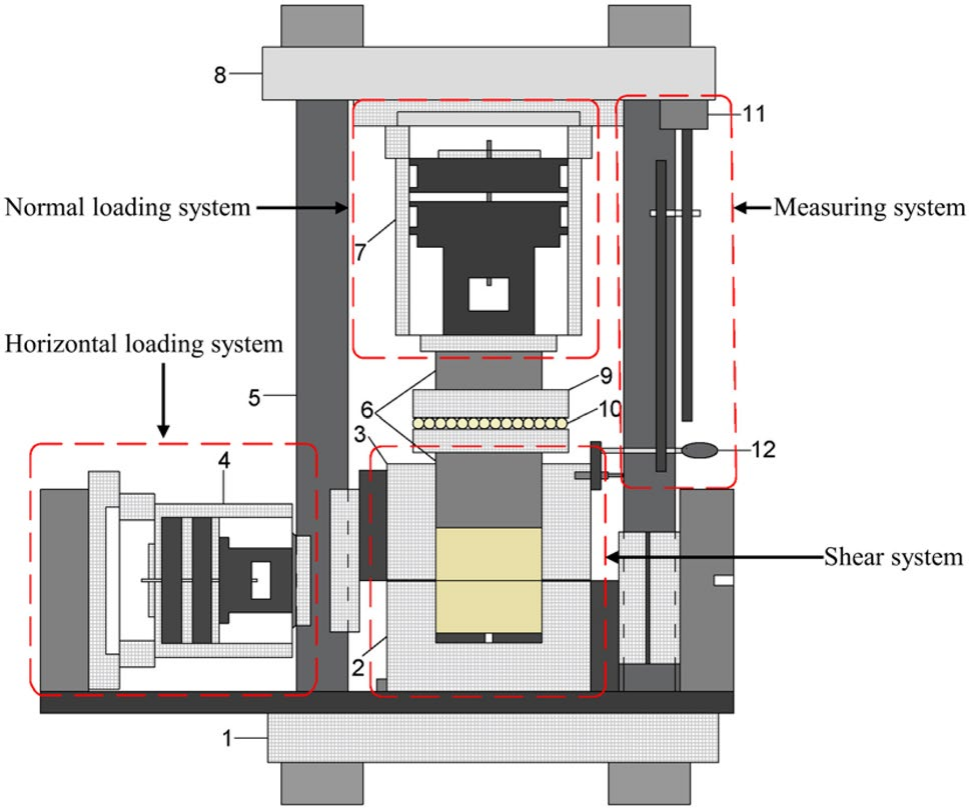
Portable rock mechanical property multifunctional test device (Chengdu University of Technology, 2004^52^). (1) Bottom frame baffle; (2) Lower shear box; (3) Upper shear box; (4) Horizontal jack loading; (5) Vertical frame baffle; (6) Force transmission device; (7) Vertical jack loading; (8) Top frame baffle; (9) Skateboards; (10) Ball bearings; (11) Magnetic stand; (12) Dial gauge.

In this study, the sandstone of Suining Formation (J_2_sn) in the Middle Jurassic system, which was collected at a depth of 10 m below the surface of a typical seismic crack slope peanut landslide in Yiliang County, Yunnan Province, was taken as the research object. The tensile stress is concentrated in the sandstone rock layer of the Huashengdi landslide, and the tensile strength of the rock layer is considerably less than its compressive strength, resulting in the fracture of the rock under stress. Rocks on both the sides are slightly displaced along the fracture surface, forming a fracture structure called joints. The joints are developed in the sandstone layer, and the fracture surface is rough, with few scratches, large joint spacing, and uneven distribution. The shear stress-displacement curves of a Jurassic red-bed sandstone intact rock sample of dimensions 100 × 100 × 100 mm [length × width × height] (Fig. 2) and a rock specimen with a flat joint surface of dimensions 100 × 100 × 50 mm (of both the upper and lower parts) (Fig. 3) were obtained through the direct shear test when the normal stress was 1, 2, and 3 MPa, respectively. The device used was a portable rock mechanical performance multifunctional instrument self-developed by Chengdu University of Technology (Fig. 4).

**Figure 5:**
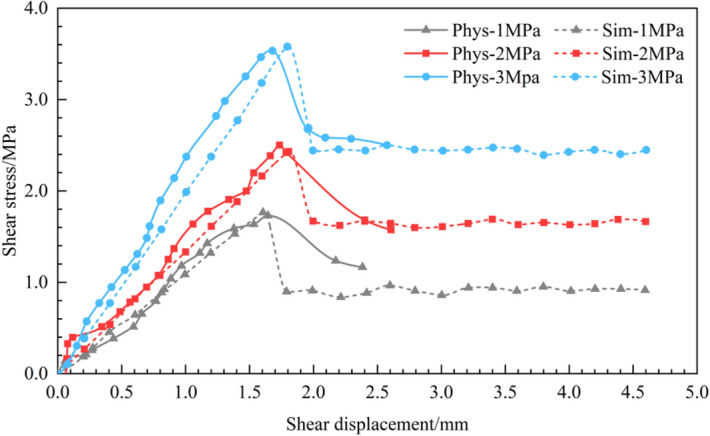
Comparison of the shear stress–displacement curves of the physical and numerical simulation tests on sandstone without a joint surface.

**Figure 6:**
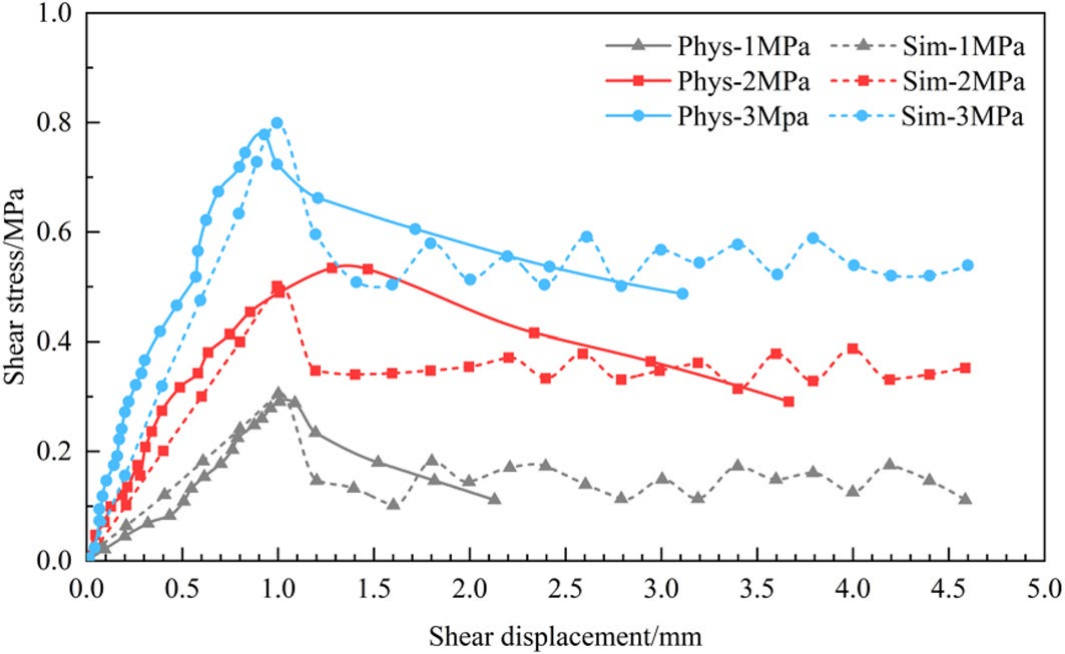
Comparison of the shear stress–displacement curves of the physical and numerical simulation tests on the flat joint surface of sandstone.

During the shearing process, the normal load remained unchanged and the shear load was gradually applied. Simultaneously, the shear and normal displacements under each shear load level were measured and recorded. Figures 5 and 6 show the shear stress-displacement curves obtained from the physical direct shear tests performed on the two rock samples. In the simulation test, the curves of the shear stress-displacement obtained from the physical direct shear tests were implemented as a calibration benchmark. By trial and error method, simulation results that were quite close to the actual test results were finally obtained (Figs. 5 and 6), the calibration parameters of which are shown in Tables 1, 2, 3.

**Table 1:** Numerical simulation test particle microscopic parameters.

Lithology	Minimum particle radius (mm)	Maximum particle radius (mm)	Particle density (g/cm^3^)	Particle contact modulus (GPa)	Normal and tangential stiffness ratio of particles	Particle friction coefficient
Sandstone	0.15	0.3	2.65	1.0	2.0	0.5

**Table 2:** Meso parameters of parallel bonding model in numerical simulation test.

Linear or parallel connection elastic modulus (GPa)	Linear or parallel bond stiffness ratio	Bonding distance/mm	Contact friction coefficient	Average contact tensile strength (MPa)	Standard deviation of contact tensile strength (MPa)	Standard deviation of contact cohesion (MPa)	Average value of contact cohesion (MPa)
1.0	2.0	0.5	0.5	10	5	5	10

**Table 3:** Meso parameters of smooth joint model in numerical simulation test.

Normal stiffness (GPa)	Normal and tangential stiffness ratio	Contact friction coefficient	Dilatancy angle (°)	Average contact tensile strength (Pa)	Standard deviation of contact tensile strength (Pa)	Average value of contact cohesion (Pa)	Standard deviation of contact cohesion (Pa)
1.0	2.0	0.5	0.0	0.0	0.0	0.0	0.0

### Experimental design and model building

**Figure 7:**
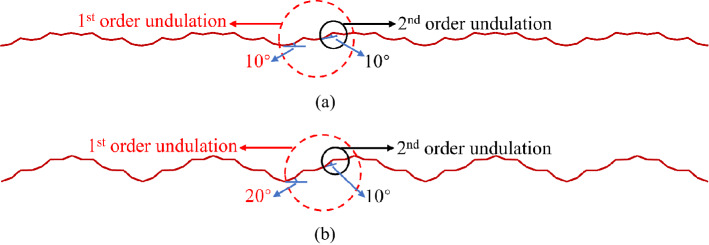
Numerical simulation model of the joint surface. (**a**) Undulation Scenario 1: the first-order initial undulant angle and the second-order undulant angle are both 10°. (**b**) Undulation Scenario 2: the first-order initial undulant angle and the second-order undulant angle are 20° and 10°, respectively.

**Figure 8:**
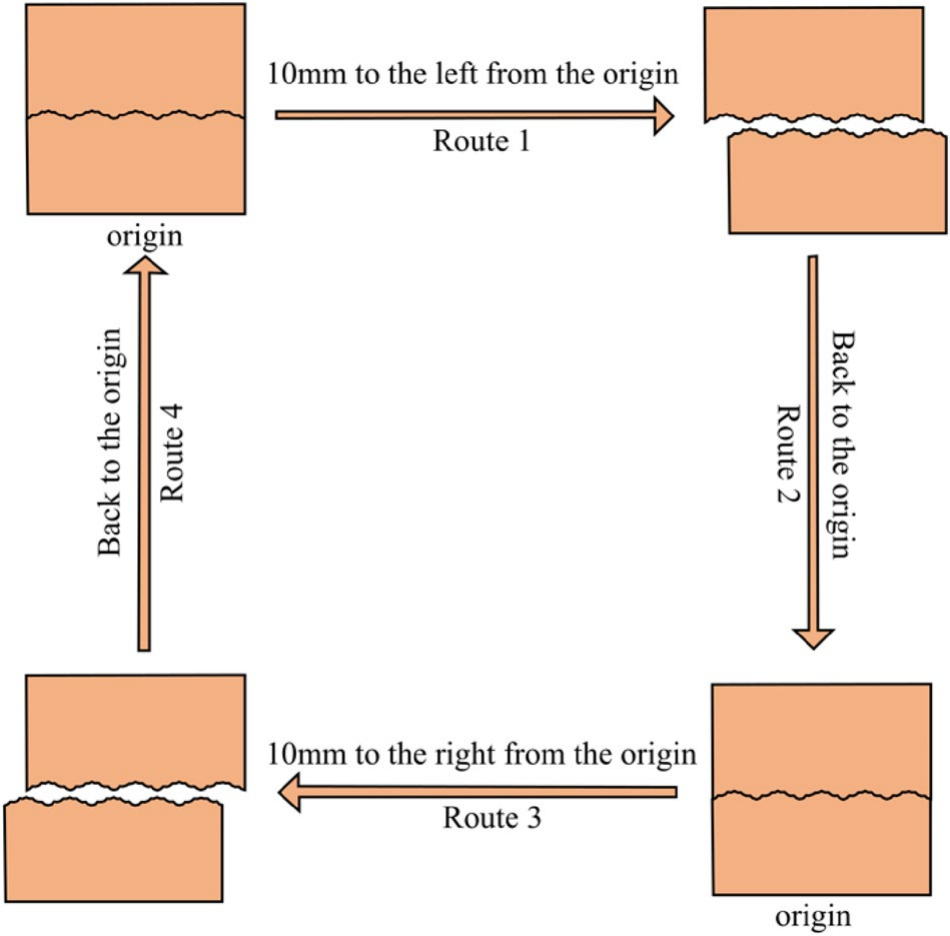
Route divisions of a single shear cycle (diagram from Dong et al.^53^).

In this study, a joint surface with regular undulations was selected. The first- and second-order undulations were considered simultaneously. In view of the undulant angles of the undulations between rock mass joint faces in nature being mostly small angles, the first-order initial undulant angle was set to be 10° and 20°, respectively, and the second-order undulant angle to be 10° (Fig. 7). The normal stress of the cyclic shear test was set to be 0.5, 1, 2, and 3 MPa, respectively. Based on the results of a string of joint surface shear tests carried out by Barton and Choubey^40^, the peak shear strength of the joint surface corresponds to a shear displacement of approximately 1% of the length of the joint surface. Additionally, the shear displacement corresponding to the residual strength is approximately 10% of the structural plane length. Thus, the shear target displacement was set to ± 10 mm—that is, 10% of the plane length. Each cycle of the cyclic shear test was divided into four steps. In the experiment, the lower shear box was stationary and the cyclic shear route was divided based on the shear displacement of the upper shear box. Route 1 shows that the upper shear box moved 10 mm to the left from the origin (0 mm). Route 2 shows that the upper shear box moved 10 mm from the maximum shear displacement on the left-hand side to the origin. Route 3 shows that the upper shear box moved 10 mm from the origin to the right. Route 4 shows that the upper shear box moved 10 mm from the maximum shear displacement on the right to the origin. (Fig. 8).

**Figure 9:**
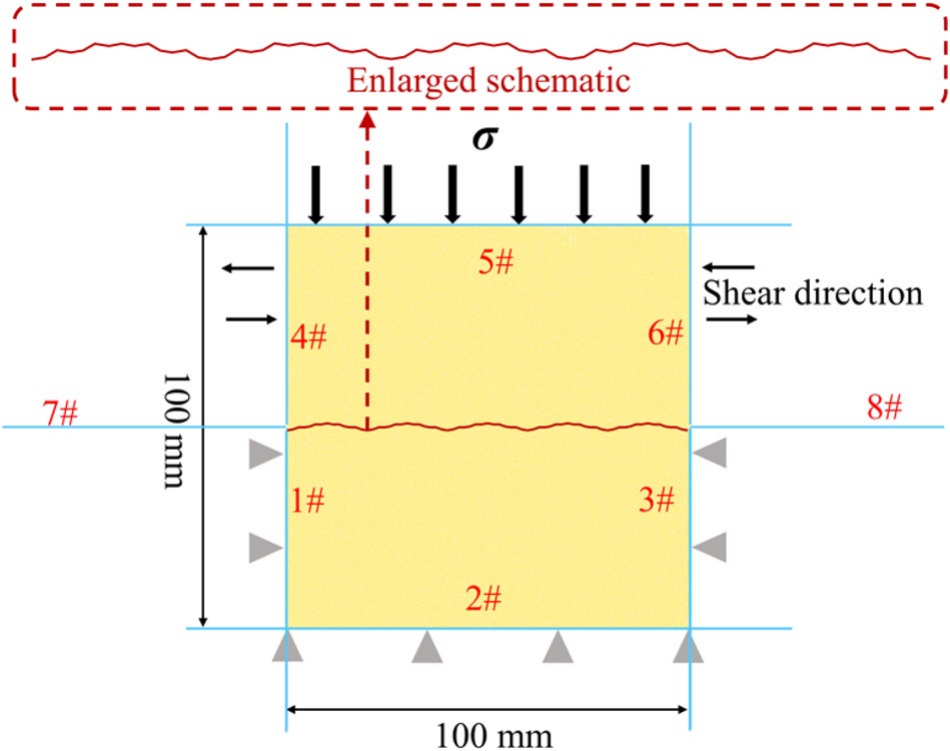
PFC numerical simulation model establishment diagram.

Based on the direct shear model of the upper and lower shear boxes established by Fu^54^ and Zhang et al.^55^ on a two-dimensional plane, eight walls were established to simulate the shear box. Walls 1#, 2#, and 3# formed the lower shear box, and walls 4#, 5#, and 6# formed the upper shear box. The upper and lower shear boxes were 50 × 100 mm in height and width, respectively. Additionally, walls 7# and 8# acted as wing walls on both sides so that the particles could not overflow (Fig. 9). The debris formed by shearing did not fall off and fill the undulations of the joint surface. The parallel bond contact model was utilized as the contact constitutive model between the rock particles in the PFC simulation—this model having the properties of tensile resistance, shear and moment effects, bond failure, and material macroscopic stiffness degradation, being more suitable for simulating rock materials^56–59^.

In addition, the smooth joint contact model could simulate a regular sawtooth rock mass joint surface. A total of 50,964 particles were randomly generated in this model, using a radius of 0.15–0.3 mm, evenly distributed, with a density of 2650 kg/m^3^, and a porosity of 0.16 (corresponding data in Table 1). Wall 5# was controlled by a servo control, a constant normal load being applied to the sample. A displacement control method was utilised to apply the shear load. The walls below the shear plane were fixed, the walls above the shear plane moving periodically at a uniform velocity of 0.5 mm/min, with an amplitude of 10 mm, stopping after five cycles.

### Analysis of evolution characteristics of shear stress

**Figure 10:**
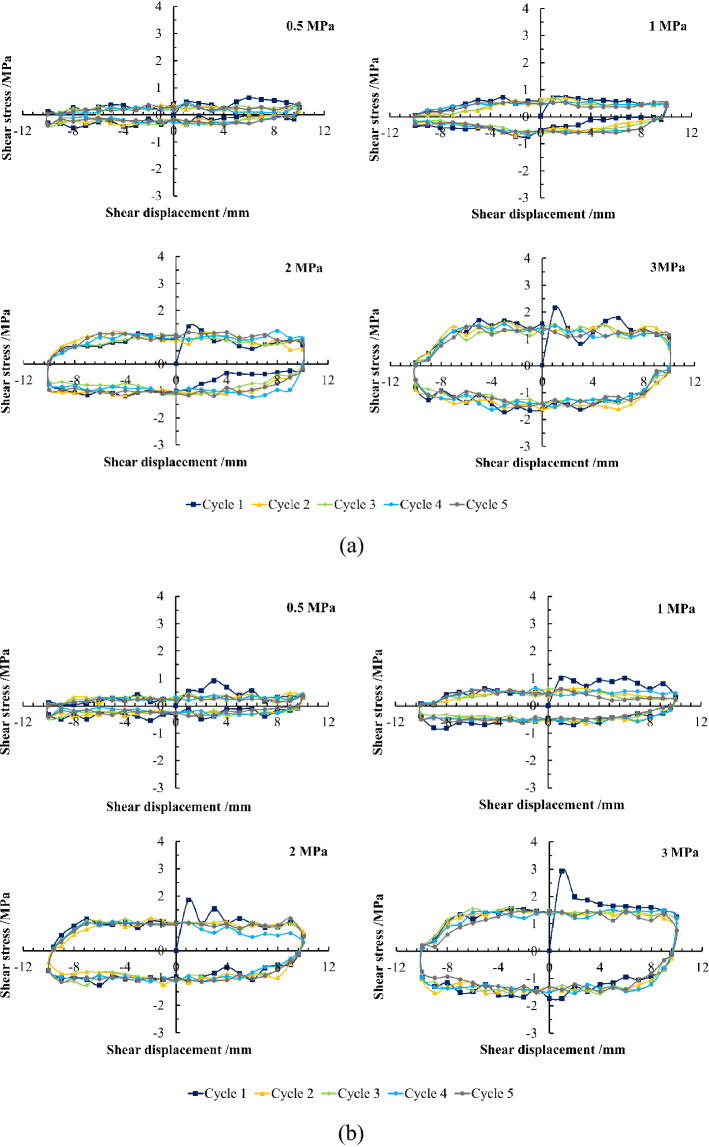
Shear stress–displacement curves of two joint surfaces under different normal stresses. (**a**) Undulation Scenario 1. (**b**) Undulation Scenario 2.

It can be seen from Fig. 10 that when the first- and the second-order initial undulant angles are both 10° (Undulation Scenario 1 as shown in Fig. 7a) for the structural plane, and the normal stress is 0.5 MPa and 1.0 MPa, separately, the shear stress–displacement curves of the joint surface under five cycles of shear all appear as slip curves, reflecting that the major shearing mechanism during cyclic shear slides along the undulations on the joint surface under low normal stress—that is, sliding failure.

**Figure 11:**
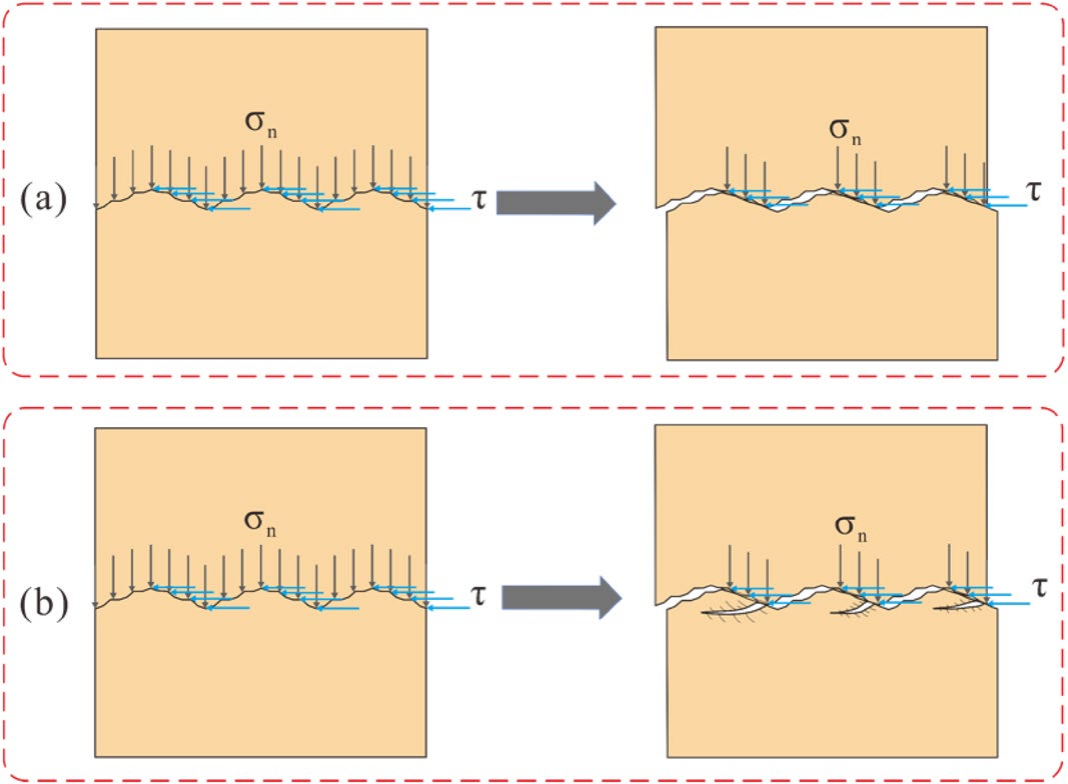
Schematic diagram of the sliding failure and shear failure modes of the shear Route 1 in first cycle of the joint surface. (**a**) Sliding failure, (**b**) shear failure. (The main difference between the two is the height at which the undulation is worn and sheared).

A schematic of the sliding failure mode is shown in Fig. 11a. During the sliding process, second-order and first-order undulations may degenerate and destroy, causing the shear plane to tend to be smooth, forming a smooth shear surface. After each shear cycle, the shear strength of the joint specimen decreases, there being little change after two or three cycles. When the normal stress is 2 and 3 MPa in Route 1 of Cycle 1, the shear stress-displacement curves of the joint surface were a peak curve, and the failure of the joint surface was mostly shear failure.

A schematic of the shear failure mode is shown in Fig. 11b. In the subsequent shear process, the main failure mode is slip failure. For the joint surface with a first-order undulant angle of 20° and a second-order undulant angle of 10° (Undulation Scenario 2 as shown in Fig. 7b), when the normal stress is 0.5 MPa, the shear process of the joint surface is dominated by slip failure, accompanied by local shear failure. Under high normal stress (1, 2, and 3 MPa), in Route 1 of Cycle 1, the shear stress–displacement curves of the joint surface are all peak curves, and the failure of the joint surface is mostly shear failure. However, under 1 MPa normal stress, Route 1 of the shear stress–displacement curve of the joint surface basically shows a peak curve, but the whole curve fluctuates greatly. The shear failure is mainly manifested by the shear along the middle and lower parts of the undulations, the residual undulations continuing to shear with increases in the shear displacement, shear rock debris filling the undulation grooves of the joint surface.

During the subsequent cyclic displacement, the degradation of second- and predominantly first-order undulations continued, but second-order undulations did not have any significant influence on the shear behavior of the joint surfaces, as they decreased after several cycles. In cycles that follow, the joint surface exhibits mainly sliding failure. Additionally, regardless of the first-order undulant angle, when the normal stress is small (0.5 MPa) for the shear failure model of Route 1 of Cycle 1, the shear curve fluctuates considerably owing to the influence of second-order undulations. This shows that when the normal stress is low, the influence of second-order undulations cannot be ignored, and its influence can be reflected in the shear curve.

Furthermore, as the normal stress increases, the slope of the elastic deformation stage increases before the shear stress reaches its peak value, that is, the shear modulus increases. After its peak value, the rate of decrease in the shear stress also increases, illustrating that the shear failure characteristics of the joint surface become increasingly evident. Additionally, under the same normal stress, the larger the undulant angle of the joint surface, the less evident the sliding failure effect of the rock mass in the shear process, the more evident the shear failure effect, and the worse the deterioration of the joint surface.

Overall, the deterioration of the joint surface shear strength under cyclic shear is mostly reflected in the first cyclic shear. In the subsequent shearing process, the shear stress-displacement curves of the joint surface are slip curves and the shear stress remains constant. This is because the joint surface is relatively straight after the undulation is sheared. During the shearing process, the sheared cuttings are filled between the structural planes as fillers, leading to friction in the structural plane in the subsequent shear process, mainly generated in three parts—that is, between the two structural planes, between the structural planes and the cuttings, and between the two sets of cuttings.

### Analysis of variation trend of dilatancy angle

**Figure 12:**
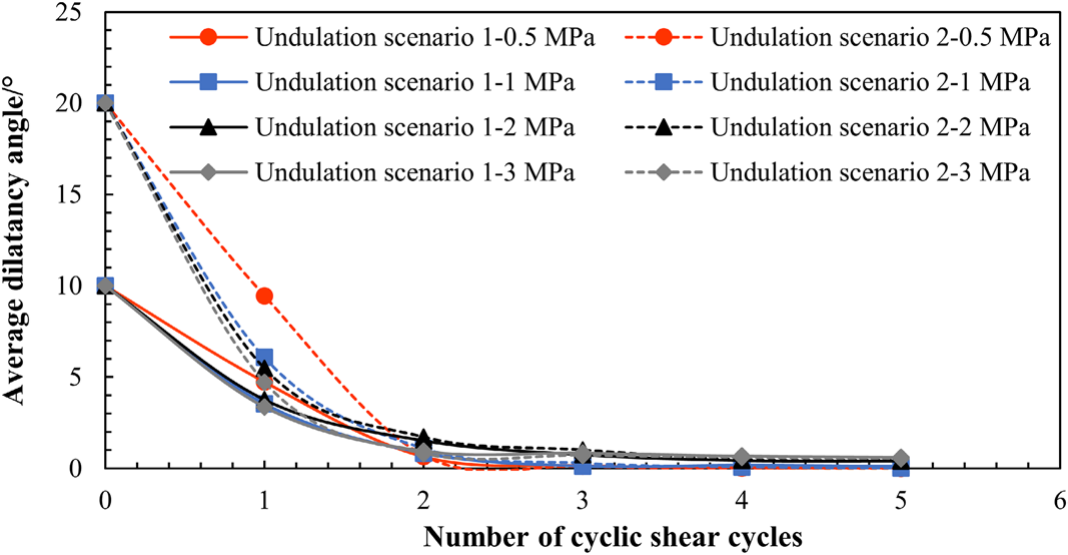
Changes in the average dilatancy angle with the number of cyclic shear cycles under different normal stresses.

The variation trend of the average dilatancy angle of the joint surface under different normal stresses with an increase in the number of cyclic shear cycles is shown in Fig. 12. The results indicate that under the same normal stress, the dilatancy angle decreases with an increase in the number of cyclic shear cycles, and when the number of cyclic shear cycles increases to a certain value, the average dilatancy angle of the joint surface increasingly became steady. Moreover, in Cycle 1, when the normal stress increases the decreasing speed of the average dilatancy angle also increases, indicating that the main shear strength deterioration under cyclic shear occurs during the first cyclic shear, and that the shear deterioration phenomenon is more evident when the normal stress increases.

The dilatancy angle can directly affect the shear strength—that is, when the dilatancy angle decreases, the shear strength decreases. Furthermore, the influence of the wear effect between structural planes should also be considered to better evaluate the changes in shear strength during cyclic shear. The first- and second-order undulation failures during cyclic shear are mainly reflected in the reduction of the dilatancy angle.

**Figure 13:**
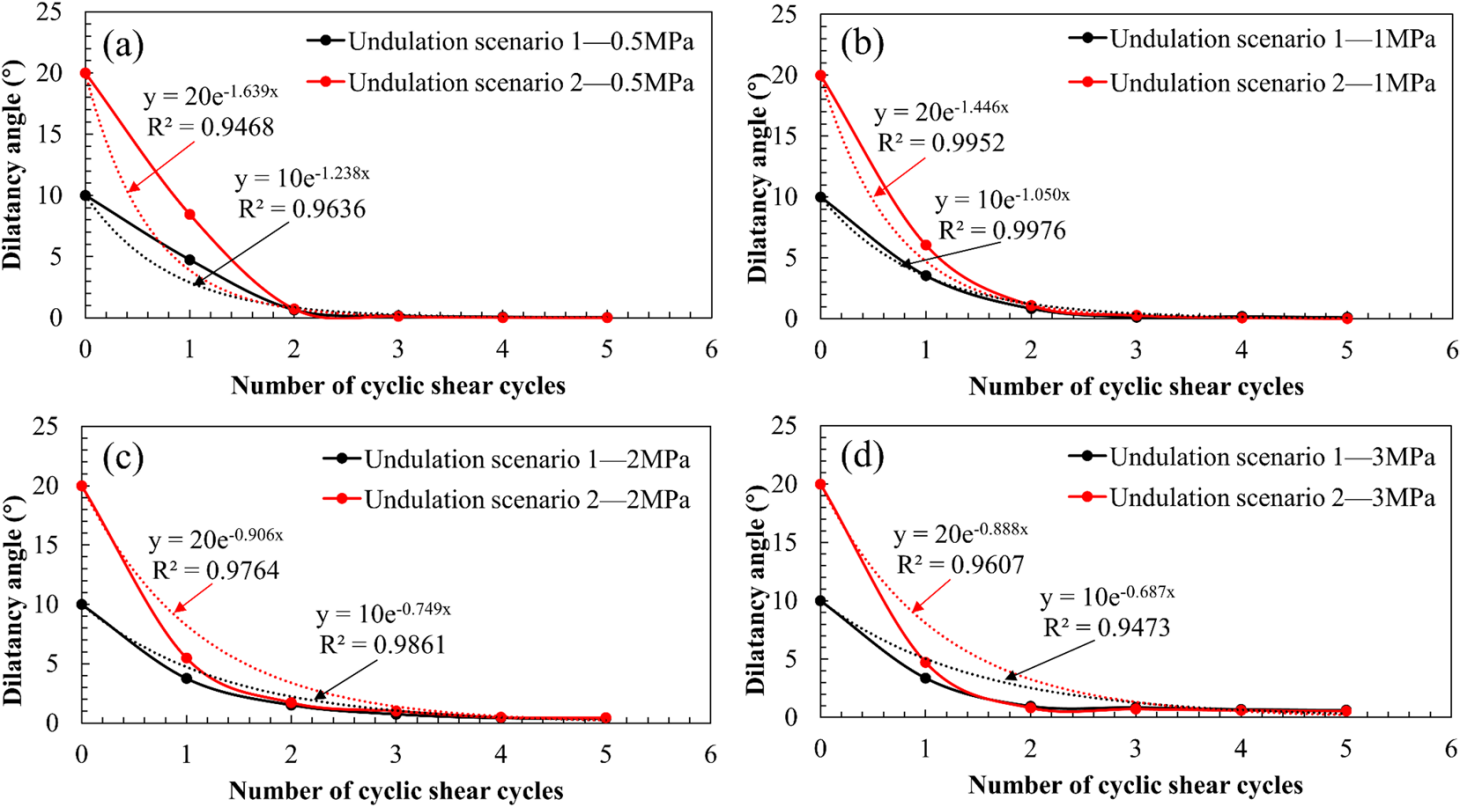
The variation trend of the dilatancy angle.

Based on the fitting results of the joint surface cyclic shear numerical simulation test, it was found that the variation trend of the dilatancy angle could be expressed by the following equation with an increase in the number of cyclic shear cycles (Fig. 13):

1 $${\alpha }_{n}={\alpha }_{0}{\mathrm{e}}^{-Bn},$$

where ${\alpha }_{n}$ denotes the average dilatancy angle of the joint surface after *n* cycles of shear, *n* denotes the number of cycles, and *B* denotes a constant related to the normal stress, which can be obtained by fitting the average dilatancy angle value of each cycle. It can be seen from Fig. 13 that the dilatancy angle of each shear cycle calculated using Eq. (1) is close to that of the entire joint surface.

**Figure 14:**
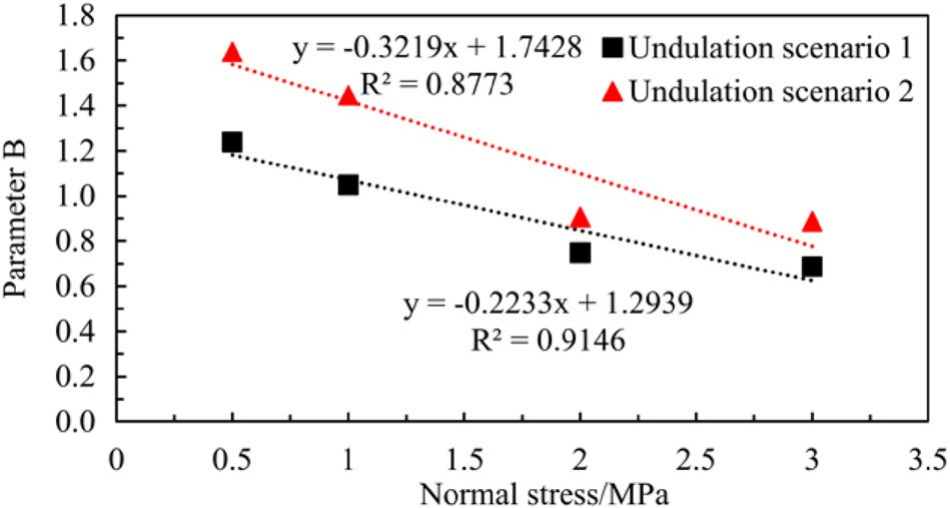
Relationship between parameter *B* and normal stress ${\sigma }_{n}$.

The relationship between parameter *B* of the joint surfaces in the two different undulation scenarios and the normal stress is shown in Fig. 14.

As shown in Fig. 14, the *B* value of the joint surface in the same undulation scenario decreases linearly with an increase in the normal stress ${\sigma }_{n}$. Simultaneously, the linear downward trend of parameter B in different undulation scenarios differs. The expression for *B* in Undulation Scenario 1 can be expressed as follows:

2 $$B=-0.2233\cdot {\sigma }_{n}+1.2939.$$

The expression of *B* in Undulation Scenario 2 can be expressed as follows:

3 $$B=-0.3219\cdot {\sigma }_{n}+1.7428.$$

The mechanical properties of the rock joint surface have the characteristics of size effect. Under complex geological conditions, large-scale rock structural plane tests are often difficult to carry out owing to factors such as capital, site, instrument, and time. Therefore, the laboratory mostly conducts small-scale joint surface physical tests, which have the advantages of simple sampling and sample preparation, convenient test operation, short cycle, and relatively low cost^60^. Based on the test results obtained from the small-scale test in the laboratory, it can reflect the large-scale situation of the project and lay a theoretical foundation for the project construction. The shear-strength deterioration formula established in this study by simulating the indoor joint surface shear test is universal to a certain extent and can be applied to a laboratory-scale test. For large-scale rock joint cyclic shear in deep underground rock mass engineering, some parameters in the calculation formula in this paper need to be re-determined, such as parameter B in the calculation formula of dilatancy angle.

## Shear strength of joint surface under cyclic shear

Barton and Choubey^40^ obtained a joint surface strength model by conducting numerous joint surface shear tests (not considering cyclic shear), as follows:

4 $$\tau ={\sigma }_{n}\cdot \mathrm{tg}\left(JRC\cdot \mathrm{lg}\frac{JCS}{\sigma }+{\varphi }_{b}\right).$$

It was also found that:

5 $${\alpha }_{d}=\frac{JRC}{2}\mathrm{lg}\frac{JCS}{{\sigma }_{n}},$$

where *JRC* denotes the roughness coefficient, *JCS* denotes the intact rock strength, and ${\alpha }_{d}$ denotes the dilatancy angle, ${\varphi }_{b}$ being the basic friction angle, and $\tau$ being the peak shear stress. The dilatancy-angle parameter in the formula can reflect the change of joint surface roughness, and, subsequently, reflect the size effect of the joint surface.

The dilatancy angles ${\alpha }_{d}$ and ${\alpha }_{n}$ have the same meaning, so they can be calculated using Eq. (1). According to Barton and Choubey^40^, the shear stress of the structural plane of *n*th cyclic shear can be defined as follows:

6 $${\tau }_{n}={\sigma }_{n}tg\left(2{\alpha }_{n}+{\varphi }_{n}\right),$$

where ${\tau }_{n}$ denotes the peak shear stress of the joint surface after *n* shear cycles, ${\alpha }_{n}$ denotes the average dilatancy angle of the joint surface after *n* shear cycles, and ${\varphi }_{n}$ denotes the basic friction angle of the joint surface after *n* shear cycles.

Based on Eq. (6), when a rock mass shears along the joint surface under a constant normal stress, the shear stress of the structural plane is mainly determined by the dilatancy angle and basic friction angle. While in the process of cyclic shear of the joint surface, the dilatancy angle and basic friction angle change dynamically with an increase in the number of cycles.

Through a cyclic shear test of a joint surface, Dong et al.^53^ found that the change trends of the basic friction angle and dilatancy angle are basically the same, and proposed a calculation method for the basic friction angle of a joint surface under cyclic shear load, which can be expressed as follows:

7 $$\frac{{\alpha }_{0}-{\alpha }_{n}}{{\alpha }_{0}-{\alpha }_{r}}=\frac{{\varphi }_{0}-{\varphi }_{n}}{{\varphi }_{0}-{\varphi }_{r}}.$$

Based on Eq. (6), the expression for the residual basic friction angle can be expressed as follows:

8 $${\varphi }_{r}=\mathrm{arctan}({\tau }_{r}/{\sigma }_{n})-2{\alpha }_{r}.$$

Based on Eqs. (7) and (8), the expression for the basic friction angle can be expressed as follows:

9 $${\varphi }_{n}=\frac{\left({\alpha }_{0}-{\alpha }_{n}\right)\left[\mathrm{arctan}({\tau }_{r}/{\sigma }_{n})-2{\alpha }_{r}\right]+\left({\alpha }_{n}-{\alpha }_{r}\right){\varphi }_{0}}{{\alpha }_{0}-{\alpha }_{r}}.$$

In Eqs. (7), (8), and (9), ${\varphi }_{n}$ denotes the basic friction angle of the surface of the *n*th cyclic shear, ${\varphi }_{0}$ denotes the basic friction angle of the surface of the joint without shear, ${\alpha }_{n}$ denotes the dilatancy angle of the *n*th cyclic shear, ${\tau }_{r}$ denotes the residual shear stress, ${\sigma }_{n}$ denotes the normal stress applied during the shear process, ${\alpha }_{0}$ denotes the initial undulant angle of the joint surface, and ${\alpha }_{r}$ denotes the residual dilatancy angle.

Based on the above analysis, the final equation for calculating the peak shear strength of the joint surface after *n* cycles under cyclic shear can be expressed as follows:

10 $$\left\{\begin{array}{l}\tau =\sigma tg\left(2{\alpha }_{n}+{\varphi }_{n}\right).\\ {\alpha }_{n}={\alpha }_{0}{e}^{-Bn}.\\ {\varphi }_{n}=\frac{\left({\alpha }_{0}-{\alpha }_{n}\right)\left[\mathit{arctan}({\tau }_{r}/{\sigma }_{n})-2{\alpha }_{r}\right]+\left({\alpha }_{n}-{\alpha }_{r}\right){\varphi }_{0}}{{\alpha }_{0}-{\alpha }_{r}}.\end{array}\right.$$

If this study is applied to cyclic shear of deep underground rock mass, the normal stress can be obtained by calculating the in-situ stress of the underground depth, the friction angle parameter can be obtained by the joint surface shear test of the borehole core sampling, and the dilatancy angle of the joint surface can be obtained by analysing the borehole photography image^61^. Therefore, they can be substituted into the Eq. (10) to analyse and predict the deterioration of shear strength of deep underground rock mass under cyclic shear load.

## Results: verification

To verify the feasibility of the proposed method, the shear stresses calculated using the proposed method were compared with those obtained from numerical simulations. Based on the test analysis results, the deterioration in the peak shear strength of the joint surface under cyclic shear was mostly reflected in the first cyclic shear. Consequently, the calculations in this study primarily considered the verification of degraded strength after the first cyclic shear.

**Figure 15:**
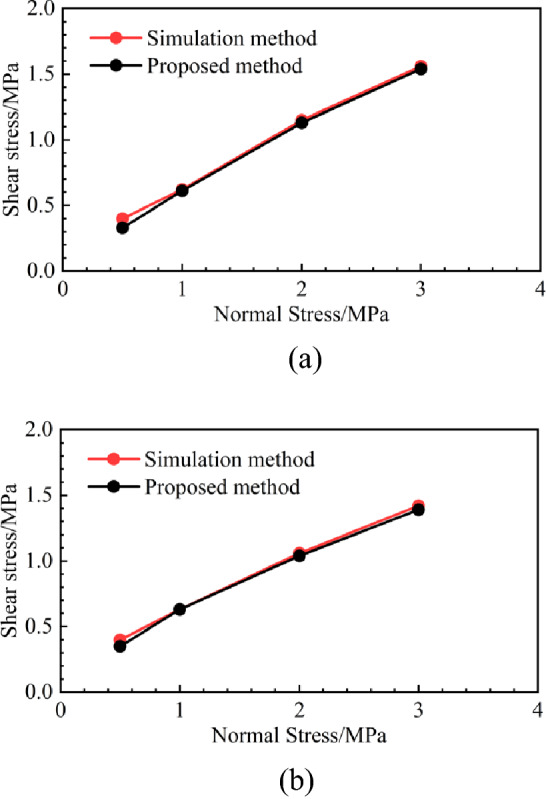
The results obtained using the simulation test and proposed method. (**a**) Undulation Scenario 1. (**b**) Undulation Scenario 2.

The dilatancy angle, basic friction angle, and peak shear strength after the first cyclic shear were calculated using Eq. (10). The calculation parameters, calculation results, and simulation test results of the proposed method after the first cyclic shear are listed in Table 4, and the simulation test and calculation results of the proposed method are shown in Fig. 15. For the structural plane in Undulation Scenario 1, when the normal stress is 0.5, 1.0, 2, and 3 MPa, the peak shear strength of simulation results is 0.40, 0.62, 1.15, and 1.56 MPa, separately; the peak shear strength of the proposed method being 0.33, 0.61, 1.13, and 1.54 MPa, separately. The errors between the simulation results and the proposed method are 17.50, 1.61, 1.74, and 1.28%, separately.

**Table 4:** Calculation parameters, calculation and simulation test results of proposed method.

Joint surface type	Normal stress (MPa)	Basic friction angle ${\varphi }_{n}$ (°)	Dilatancy angle ${\alpha }_{n}$ (°)	Simulation test results (MPa)	Calculation results of the proposed method (MPa)	Errors between the two methods (%)
Undulation Scenario 1	0.5	21.72	5.03	0.40	0.33	17.5
	1.0	26.32	4.72	0.62	0.61	1.61
	2.0	26.41	3.50	1.15	1.13	1.74
	3.0	25.33	2.90	1.56	1.54	1.28
Undulation Scenario 2	0.5	24.66	8.23	0.40	0.35	12.5
	1.0	25.72	8.08	0.63	0.63	0.00
	2.0	25.16	4.71	1.06	1.04	1.89
	3.0	23.31	3.88	1.42	1.39	2.11

For the joint surface in Undulation Scenario 2, when the normal stress is 0.5, 1.0, 2, and 3 MPa, the peak shear strength of the simulation results is 0.40, 0.63, 1.06, and 1.42 MPa, separately; the peak shear strength of the proposed method results being 0.35, 0.63, 1.04, and 1.39 MPa, separately; The errors between the simulation results and the proposed method are 12.50, 0.00, 1.89, and 2.11%, respectively. When the normal stress is 0.5 MPa, the calculation errors of the joint surface in Undulation Scenario 2 are greater than 3%, and the error is a little large. The reason for this is explained in detail in “Comparisons”. Furthermore, the error in the other calculation results is within 3%, which demonstrates the feasibility of the proposed method.

## Discussion

### Previous studies

In order to prove the effectiveness of the peak shear strength models proposed in this article, the numerical simulation results were compared with the values calculated by the proposed model as well as the models of Barton and Choubey^40^ and Homand et al.^18^. The calculation method for peak shear strength by Barton and Choubey^40^ is discussed in “Shear strength of joint surface under cyclic shear”. In this paper, when the Barton method was applied, the basic friction angle was the residual friction angle of the joint surface after *n* cycles of shear, and the dilatancy angle was the average dilatancy angle of the joint surface after *n* cycles of shear, used to calculate the deteriorated shear strength after each cyclic shear. The average dilatancy angle (${\alpha }_{a}$) and residual friction angle (${\varphi }_{r}$) of the joint surface after *n* cycles of shearing were obtained by numerical simulation shear tests, which differed from the parameters calculated using the proposed method.

Here, the Homand shear strength model^18^ is introduced briefly. Homand et al.^18^ used cement as a similar material to prepare regular undulating joints and carried out ten shear cycle tests. The step length of the serrated undulation of the joint surface was 25 mm, the height of the first-order undulation was 2 mm, the maximum displacement in one direction of each cycle was 10 mm, and the total shear displacement was 400 mm. A new computer-controlled three-dimensional shear apparatus (CC3DSM) was used for the test. The tests were conducted at six normal stress levels of 0.5–5 MPa. The surface wear characteristics of artificial regular wavy joints before and after shearing were studied. Using the defined surface roughness degradation index, the changes in the surface wear characteristics and degradation index of the joints with the number of shear cycles and their relationship with the normal stress were analysed. Based on these theoretical analyses, Homand et al.^18^ proposed a constitutive model for predicting the peak shear strength of joint surfaces under cyclic shear conditions, based on Barton’s formula, as follows:

11 $$\tau ={\sigma }_{n}tan\left({\varphi }_{b}+{\alpha }_{p}\right),$$

12 $${\alpha }_{p}=2{\theta }_{s}^{0}\mathrm{exp}\left(-b{\sigma }_{n}\right),$$

where ${\sigma }_{n}$ denotes the normal stress, ${\varphi }_{b}$ denotes the basic friction angle, ${\alpha }_{p}$ denotes the peak dilatancy angle, and ${\theta }_{s}^{0}$ denotes the initial three-dimensional average angle before the cutting process. For the regular serrated joint surface, ${\theta }_{s}^{0}={\alpha }_{0}$, *b* is a constant that depends on the uniaxial compressive strength of the intact specimen (${\sigma }_{c}$), the initial roughness (${DR}_{r}^{0}$), the apparent surface anisotropy (${k}_{a}$), and the maximum shear displacement (${u}_{max}$), related to the number of cycles. The constant *b* can be expressed as follows:

13 $$b=\frac{1}{{\sigma }_{c}}\left(\frac{{u}_{0}^{2}}{{a}_{0}{u}_{max}}+\frac{{k}_{a}}{{DR}_{r}^{0}}\right),$$

where ${a}_{0}$ denotes the maximum amplitude of the serrated joint surface, and ${u}_{0}$ denotes the maximum positive shear displacement (or the maximum displacement of monotonic shear displacement). For surfaces with a serrated plane, ${k}_{a}=0$. The values of the calculation parameters in the formula used in this study (when using the Homand method) are listed in Table 5.

**Table 5:** Calculation parameters in the Homand formula for cyclic shear strength of joints.

${\sigma }_{n}$ (MPa)	${\varphi }_{b}$	${\sigma }_{c}$ (MPa)	${a}_{0}$ (mm)	${u}_{0}$ (mm)	${k}_{a}$	${\theta }_{s}^{0}$	${u}_{max}$ (mm)
0.5/1.0/2.0/3.0	25.3°	40.6	1.76	10	0	10°/20°	(*n* + 1)*${u}_{0}-3/4{u}_{0}$

*n is the number of cyclic shear cycles.

### Comparisons

**Figure 16:**
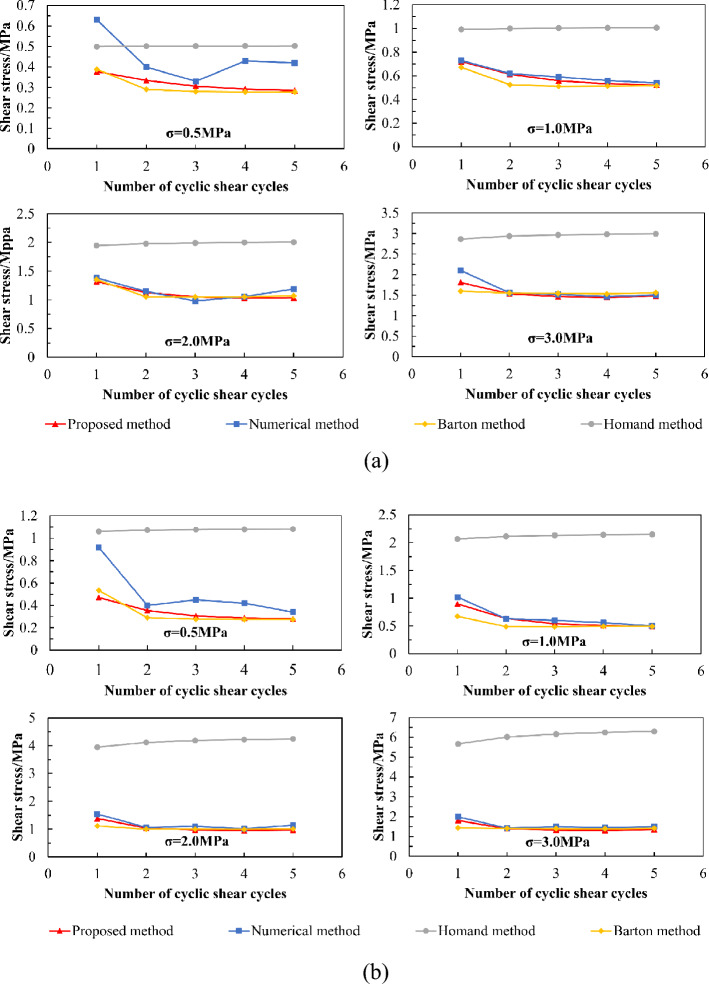
Comparison of calculation results of cyclic shear strength using four methods. (**a**) Undulation Scenario 1. (**b**) Undulation Scenario 2.

Comparison of the peak shear strength results obtained using the numerical simulation method, the method proposed in this study, the Barton method, and the Homand method for five cycles of shear is shown in Fig. 16.

It can be seen from Fig. 16 that under higher normal stress (1, 2, and 3 MPa), the calculated peak shear strength of the joint surfaces with different undulant angles gradually decreases with an increase in the number of cycles, the decreasing trend slowing down step by step, which is in fine agreement with the numerical simulation results. A large number of shear-test results indicate that the difference of undulating characteristics in different directions and the size of normal stress on the joint surface are the main factors that make the shear mechanical behaviour of the joint surface anisotropic^62^. Zhou et al.^63^ demonstrated that there is a positive correlation between the anisotropy of joint surface roughness and peak shear strength, and the increase of normal stress weakens the anisotropy of shear mechanical behaviour of the joint surface. The morphological characteristics of joint surfaces are important factors affecting the shear behaviour of joint surfaces, and there is a certain relationship between the anisotropy of shear mechanical behaviour of joint surfaces and the anisotropy of morphological characteristics. With the increase of cyclic-shear times, the undulations of sandstone joint surface are sheared during the shear process, and the debris fills the voids between the undulations. The sliding surface becomes smooth, reducing the roughness of the joint surface, weakening the anisotropy, and slowing down the decline of shear strength.

Additionally, the calculated values of the proposed method are close to those of the Barton method. However, the degree of fitting of the simulation results to the calculation results obtained using the proposed method is better than those obtained using the Barton method. At the same time, the calculated values of the proposed method are lower than those of the numerical simulation. This is because the proposed method considers the ideal state—that is, it does not consider the influence of rock cutting fillings on the shear strength of the joint surface in the cyclic shear process. In fact, during the shearing process, because of the wing walls on both sides of the model, the sheared rock cuttings cannot fall off and are filled between the joint surfaces as fillers, causing friction in the joint surfaces in the subsequent shearing processes, which occurs in three parts—that is, between the two joint surfaces, between the joint surface and the rock cuttings, and between the two groups of rock cuttings—resulting in a large basic friction angle between the joint surfaces. Consequently, the numerical simulation results are slightly larger than those calculated using the proposed method.

Under a lower normal stress (0.5 MPa), there is a large deviation between the calculated value of the proposed method and the numerical simulation results. Huang et al.^14^ carried out a cyclic shear test of joint surfaces in nature and concluded that second-order undulations cause the peak shear stress in the first cycle to be considerably higher than that in the subsequent cycles under low normal stress. However, the undulation is sheared along the middle and lower parts during the first shear cycle under high normal stress, and the subsequent shear is dominated by friction and sliding between the joint surface and rock debris, which is hardly affected by the second-order undulation. Consequently, because the influence of second-order undulation is not considered in the proposed method under low normal stress, there may be some deviation in the results of the cyclic shear strength of low normal stress joints using the proposed method.

Based on Fig. 16, the results of the Homand method are greater than those of the numerical results and the other two methods. This is because when calculating the cyclic shear strength degradation of the serrated joint surface, the value of the apparent surface anisotropy (${k}_{a}$) in the Homand method is 0. The degradation effect of the joint anisotropy and roughness with cyclic shear cannot be considered, and the Homand method does not consider the degradation effect of the basic friction angle with cyclic shear. Consequently, the results using the Homand method are slightly greater. Only the maximum shear displacement (${u}_{max}$) in the parameters that affect the peak dilatancy angle (${\alpha }_{p}$) in the formula is related to the number of cycles, and the ${u}_{max}$ that varies with the number of cycles has a weak effect on the peak shear dilatancy angle ${\alpha }_{p}$. This results in a slight change in the peak dilatancy angle (${\alpha }_{p}$) with the number of cycles, indicating that the peak shear strength does not change much with an increase in the number of cycles.

The shear strength varies significantly owing to different rock materials^64^. This paper mainly focuses on the mechanical behaviour of sandstone, and the proposed formula is only based on the cyclic shear test and verification of the joint surface of sandstone rock mass. Therefore, the coefficients of the proposed formula may only be applicable to the calculation of sandstone or mudstone rock mass. For other types of rock mass, the values of relevant parameters must be determined again through tests. In the subsequent research, it is necessary to study the degradation law of peak shear strength in the process of cyclic shear of the joint surface of different lithologic rock masses, study and analyse as many test data as possible, and improve the proposed formula, especially the relevant parameters in the formula, so as to adapt to the joint surface of a wide range of rock materials as much as possible.

## Conclusions

In this study, based on the numerical simulation test results of cyclic shear using PFC numerical simulation software considering the first- and second-order undulations, the variation laws of the dilatancy angle and shear stress with changes in the number of cyclic shear cycles were obtained. A calculation formula for the peak shear strength of the joint surface under cyclic shear load considering the degradation of the dilatancy angle and basic friction angle was determined. The following conclusions could be drawn:

1. The greater the normal stress and undulant angle, the more serious the deterioration of the joint surface under cyclic shear load, the deterioration of the peak shear strength of the joint surface being mainly reflected in the first cyclic shear. The dilatancy angle decreased with an increase in the number of cyclic shear cycles. When the number of cyclic shear cycles increased to a certain value, the average dilatancy angle of the joint surface tended to gradually stabilize.
2. To verify the effectiveness and accuracy of the method proposed in this study, the peak shear strength of the specimen under five cycles of cyclic shear was calculated using the established cyclic shear strength formula and compared with the results of the numerical simulation, Barton method, and Homand method. The results showed that the proposed method generated results close to those of the numerical simulation and could predict the degradation law of the joint surface peak shear strength in the process of cyclic shear. The coefficient of the proposed formula may only be applicable to sandstone, and the relevant parameters for other rock masses must be re-determined through tests.

## Data Availability

The data that support the findings of this study are available on request from the corresponding author.

## List of symbols

${\alpha }_{n}$: Dilatancy angle of the n-th cyclic shear
*n*: Number of shear cycles
*B*: A constant related to the normal stress
$${\sigma }_{n}$$: Normal stress
τ: Peak shear stress
$JRC$: Joint roughness coefficient
$JCS$: Intact rock strength
$${\alpha }_{d}$$: Dilatancy angle
${\varphi }_{b}$: Basic friction angle
${\tau }_{n}$: Shear stress of the joint surface after n shear cycles
${\varphi }_{r}$: Residual basic friction angle
${\varphi }_{n}$: Basic friction angle of the surface of the nth cyclic shear
${\varphi }_{0}$: Basic friction angle of the surface of the joint without shear
${\tau }_{r}$: Residual shear stress
${\alpha }_{0}$: Initial undulant angle of the joint surface
${\alpha }_{r}$: Residual dilatancy angle
${\alpha }_{a}$: Average dilatancy angle
${\alpha }_{p}$: Peak dilatancy angle
${\theta }_{s}^{0}$: Initial three-dimensional average angle before the cutting process
*b*: A constant that depends on the uniaxial compressive strength of the intact specimen
${\sigma }_{c}$: Uniaxial compressive strength of the intact specimen
$${DR}_{r}^{0}$$: Initial roughness
${k}_{a}$: Apparent surface anisotropy
${u}_{max}$: Maximum shear displacement
${a}_{0}$: Maximum amplitude of the serrated joint surface
${u}_{0}$: Maximum positive shear displacement
